# Digitalisierung und Sorgebeziehungen von Alleinlebenden im Alter: eine kritische Analyse politischer Dokumente

**DOI:** 10.1007/s00391-024-02309-0

**Published:** 2024-06-03

**Authors:** Rafaela Werny, Marie Reich, Miranda Leontowitsch, Frank Oswald

**Affiliations:** 1 Interdisziplinäre Alternswissenschaft (IAW), Fachbereich Erziehungswissenschaften, Goethe-Universität Frankfurt, Theodor-W.-Adorno-Platz, 60629 Frankfurt am Main, Deutschland; 2 Graduiertenkolleg Doing Transitions, Goethe-Universität Frankfurt, Theodor-W.-Adorno-Platz 1, IKB-Gebäude, Postfach 3, 60323 Frankfurt am Main, Deutschland

**Keywords:** Soziales Netzwerk, Digitale Transformation, Care, Sozialpolitik, Kritische Dokumentenanalyse, Social network, Digital transformation, Care, Social policies, Critical document analysis

## Abstract

Die Digitalisierung aller Lebensbereiche transformiert das gesellschaftliche und soziale Miteinander. Es entstehen neue kulturelle Vorstellungen von Sorgebeziehungen, die ältere Menschen vor Herausforderungen stellen können. Diese gelten insbesondere für Alleinlebende im Alter, die seltener digitale Geräte benutzen und bei der Bewältigung von zunehmend digitalisierten Aufgaben (Termine, Buchungen, finanzielle Angelegenheiten) auf Unterstützung angewiesen sind. Der vorliegende Artikel untersucht das Verhältnis von Digitalisierung und Sorgebeziehungen von alleinlebenden älteren Menschen mithilfe einer kritischen Dokumentenanalyse nach Bacchi. Dieses Vorgehen ermöglicht es, das Verständnis hinter den Begriffen Alleinleben, Sorgebeziehungen und Digitalisierung in Bezug auf ältere Menschen einzeln und im Zusammenspiel in den Blick zu nehmen sowie Leerstellen sichtbar zu machen. Die Analyse von Sozialgesetzgebung und politischen sowie zivilgesellschaftlichen Dokumenten führt zu zwei zentralen Ergebnissen: Zum einen wird eine individuelle Verantwortungszuschreibung im Hinblick auf die Versorgung sowie den Zugang und Umgang mit Digitalisierung an ältere Menschen und ihre Beziehungsnetzwerke deutlich. Diese Individualisierung erscheint besonders signifikant, da die Digitalisierung als Lösungsansatz für gesamtgesellschaftliche Probleme, wie den demografischen Wandel oder den Pflegekräftemangel, präsentiert wird. Zum anderen zeigt die Analyse, dass in den Dokumenten, die Handlungsrahmen schaffen, Digitalisierung im Alter nicht thematisiert wird. Diese fehlende Fokussierung hat zur Folge, dass Handlungsstrategien schwach ausfallen.

## Abstract (en)

Digitalization is transforming social life and relationships. New cultural ideas of care, cooperation and reciprocity are emerging that can create challenges for older people. These are particularly poignant for older people living alone, who use digital devices less frequently and rely on support to manage the increasing number of digital tasks (appointments, bookings, financial matters). This article explores the relationship between digitalization and care relationships among older people living alone using a critical document analysis approach according to Bacchi. This approach makes it possible to look at the understanding behind the terms living alone, care relationships, and digitalization, each in relation to older people and in interaction as well as to render gaps visible. The analysis of laws and social policies as well as of political and civil society orientated documents leads to two key findings: Firstly, an individual responsibility to deal with needs arising from gaining access to and handling of digitalization is expected of older people and their social networks. This is striking as digitalization is presented as a means of dealing with the large-scale social challenges, such as demographic change and shortage of care staff. Secondly, the analysis shows that legal documents that set out the structure for policies do not focus on digitalization in later life. With older people not being a target group of policy of digitalization, fewer strategies are put into practice.

## Hintergrund und Fragestellung

Die fortschreitende Digitalisierung in allen Lebensbereichen und über alle Altersgruppen hinweg erhöht den Druck auf ältere Menschen, digitale Geräte zu nutzen, um anschlussfähig zu werden und zu bleiben. Dies betrifft sowohl die öffentliche und gesundheitliche Versorgung als auch die privaten Sorge- und Beziehungsnetzwerke. Vor dem Hintergrund einer stetig wachsenden Gruppe alleinlebender Menschen im Alter, die befähigt werden sollen, möglichst lange selbstständig zu Hause zu leben, ist die Auseinandersetzung mit ihrem digitalen Sorge- und Beziehungsnetzwerk von großem empirischem, politischem und gesellschaftlichem Interesse. Dieser Beitrag widmet sich der Frage, wie die Verbindung von Sorgebeziehungen^1^, Digitalisierung und Alleinleben im Alter politisch organisiert, reguliert und zivilgesellschaftlich debattiert wird. Um diese Frage zu beantworten, wurde eine kritische Dokumentenanalyse nach Bacchi [1] durchgeführt.

## Studiendesign und Untersuchungsmethoden

Im Rahmen der Analyse wurden politische Dokumente wie Gesetztestexte, nationale Strategien, von Ministerien in Auftrag gegebene Berichte und Stellungnahmen von zivilgesellschaftlichen Organisationen, die die Themen ältere Menschen, Sorgebeziehungen und Digitalisierung zum Thema haben, ausgewählt (Tab. 1). Die Dokumente lassen sich in zwei Typen aufteilen: Sozialgesetzgebung als ordnungspolitische Richtungsentscheidung des Gesetzgebers und politische und zivilgesellschaftliche Berichte, Strategien und Positionspapieren, die sich mit konkreten Problemlagen bzw. Herausforderungen auseinandersetzen. Diese beiden Typen ermöglichen es auf zwei Ebenen, politische und zivilgesellschaftliche Ausgestaltung sowie abgeleitete Forderungen zu verhandeln.

**Tab. 1:** Übersicht der analysierten politischen Dokumente

Titel	Jahr
Sozialgesetzbuch XI – Soziale Pflegeversicherung (SGB XI) [5]	1994/2014
Sozialgesetzbuch V – Gesetzliche Krankenversicherung (SGB V) [15]	1988/2021
Sozialgesetzbuch XII – Sozialhilfe (SGB XII)	2003/2021
Siebter Altenbericht: Sorge und Mitverantwortung in der Kommune – Aufbau und Sicherung zukunftsfähiger Gemeinschaften [3]	2016
Siebter Altenbericht: Stellungnahme der Bundesregierung [20]	2016
Achter Altersbericht: Ältere Menschen und Digitalisierung [4]	2020
Achter Altersbericht: Stellungnahme der Bundesregierung [9]	2020
Achter Altersbericht: Stellungnahme der Bundesarbeitsgemeinschaft der Seniorenorganisationen (BAGSO) [10]	2020
Achter Altersbericht: Stellungnahme Fachbeirat Digitalisierung und Bildung für ältere Menschen (DigiBÄM) [11]	2020
Positionspapier: Ältere Menschen und digitale Technologien in der Zeit der Corona-Pandemie	2020
Positionspapier: Soziale Ungleichheit durch Zugang für alle reduzieren – Chance vertan [12]	2020
Positionspapier: Ältere Menschen in der digitalen Welt [16]	2016/2017
Digitalpakt Alter [19]	2021
Impulspapier für den Koalitionsvertrag der Bundesregierung Deutschland 2021	2021
Koalitionsvertrag der Bundesregierung Deutschland 2021 [18]	2021
Positionspapier: Den digitalen Wandel im höheren Lebensalter in Deutschland gestalten [17]	2021
Digitalisierung gestalten. Umsetzungsstrategie der Bundesregierung [7]	2021
Strategie Digitales Hessen: intelligent, vernetzt, für alle [8]	2016
Digitales Hessen: Wo Zukunft zuhause ist [6]	2021
Smart City Frankfurt	2021

Mithilfe des von Bacchi entwickelten Ansatzes „What’s the Problem Represented to be?“ (WPR) wurden die politischen Dokumente (siehe Tab. 1) einer kritischen Dokumentenanalyse, die Machtdynamiken, Vorannahmen und gesellschaftliche Diskurse sichtbar macht, unterzogen [1]. Neben der Aufdeckung problematischer Darstellungen zielt Bacchis Ansatz darauf ab aufzuzeigen, wie z. B. ältere Menschen wahrgenommen und adressiert werden. So kann gezeigt werden, in welchen Zusammenhängen manche Gruppen sichtbar gemacht, während andere marginalisiert werden. Dazu wird zunächst analysiert, was in den Dokumenten als „problematisch“ und somit politisch relevant angesehen wird. Aufbauend darauf wird herausgearbeitet, wie soziale Akteur*innen politische Argumente handhaben, und wie infolgedessen bestimmte Gruppen von Bürger*innen (z. B. ältere Menschen) als investitionswürdig identifiziert und somit zu „Zielpopulationen“ [2, S. 334] von Sozialpolitik gemacht werden. So kann aufgezeigt werden, wie Politik bestimmte Gruppen in politischen Dokumenten marginalisiert, welche Annahmen über diese Gruppen die Politik bestimmen, welche Aspekte dabei problematisiert werden, und wie die politischen Lösungen dieser Probleme durch die Vorannahmen geprägt sind. Im Analyseprozess wurden die Dokumente zunächst einzeln auf die Verhandlung der Begriffe Alleinleben, Alter, Sorgebeziehungen und Digitalisierung kodiert und dann auf ihr Zusammenspiel der Begriffe hin untersucht. In einem zweiten Schritt wurden die Begriffsverhandlung zwischen den Dokumenten verglichen, um Übereinstimmungen, Widersprüche und Konfliktfelder sichtbar zu machen. Diese Zwischenergebnisse ergaben zwei Kategorien, die im Ergebnisteil vorgestellt werden.

## Ergebnisse

### Individualisierung von Sorge(‑Beziehungen) und digitale Kompetenzen älterer Menschen

Alter(n) wird in der Mehrzahl der analysierten Dokumente im Rahmen des demografischen Wandels als gesamtgesellschaftliches Problemfeld thematisiert. Die Herausforderungen, die eine alternde Gesellschaft mit sich bringt, prägen die Herangehensweise an die Themen Digitalisierung, Alleinleben und Sorgebeziehungen. So wird Alleinleben im Alter, unabhängig vom Dokumententyp, ausschließlich bezogen auf das Ziel eines möglichst langen und selbstständigen Verbleibens in der eigenen Wohnung thematisiert [3, S. 23, 106, 4, S. 64, 5, § 3]. Nach diesem Ziel richtet sich auch die nationale und föderale Pflegepolitik. Sie beruht auf § 3 der Inanspruchnahme informeller familiärer Netzwerke für die Pflege älterer Menschen, bevor formelle Pflege finanziert wird [5].

Die politische Strategie, dem erhöhten Sorgebedarf einer alternden Gesellschaft gerecht zu werden, beruht nicht auf einer Verantwortungsübernahme durch die Gesamtgesellschaft oder den Staat, sondern verbleibt in einer Verantwortungszuschreibung an individuelle Netzwerke. Wo familiäre Netzwerke die Unterstützung nicht (mehr) leisten können, sollen Nachbar*innen, Bekannte und andere ältere Personen als ehrenamtliche Helfer*innen herangezogen werden [3, S. 20, 241 f.]. Agile ältere Menschen werden so auch als Ressource für unbezahlte Arbeit im Sorgebereich betrachtet.

Auch die Digitalisierung wird als ein adäquates Mittel dargestellt, mit dem den Auswirkungen des demografischen Wandels, dem steigenden Pflegebedarf sowie den wenig verfügbaren Pflegekräften begegnet werden soll. Dabei lassen sich unterschiedliche Perspektiven ausmachen: die Kommission des Achten Altersberichts verweist auf die hohe Bereitschaft und Motivation älterer Menschen zur Nutzung digitaler Geräte, die auf ihre Bedürfnisse abgestimmt sind und beispielsweise ihren Alltag erleichtern. Aufbauend darauf betonen sie das große Potenzial der Digitalisierung und der technischen Entwicklung für das Leben älterer Menschen. Voraussetzung dafür ist, dass die technischen und digitalen Geräte in den Alltag älterer Menschen gut eingebettet sind, ihnen und ihren Angehörigen ausreichend Informationen, Beratung und Unterstützung zur Verfügung stehen und sie in die technische Entwicklung eingebunden werden. Ausgehend davon könnten technische Systeme Sorgebeziehungen unterstützen und entlasten und so Zeit und Raum für die emotionalen Aspekte von Pflege schaffen [4, S. 55]. Dieses kompetenzorientierte Altersbild lässt sich in der Stellungnahme der Bundesregierung weniger stark ausmachen. Hier dominiert die Vorstellung von älteren Menschen als eine Bevölkerungsgruppe, die von dem Nutzen bestimmter Technik überzeugt und motiviert werden muss, um den demografischen Wandel und Pflegenotstand zu kompensieren und Kosten zu sparen [9]. Bedürfnisse älterer Menschen geraten so an den Rand, und ein defizitorientierter Blick auf das Alter wird aktualisiert. Hier zeigt sich ein Dilemma: Auf der einen Seite soll älteren Personen Handlungsfähigkeit zugesprochen werden; auf der anderen Seite darf dies nicht zu mangelnden Formen der Unterstützung oder Nichternstnehmen ihrer Bedürfnisse führen.

Die Darstellung der gesellschaftlichen und sozialen Digitalisierung wird als Chance für Autonomie und soziale Teilhabe im Alter verhandelt und auf der individuellen Ebene eng mit der Lösung gesamtgesellschaftlicher Probleme verbunden. Während zivilgesellschaftliche Organisationen und auch die Sachverständigenkommission des Achten Altersbericht den Bedarf an Information, Beratung und Unterstützung betonen, wird in Berichten der Bundes- und Landesregierung^2^ die Verantwortung auf den Einzelnen und seine Beziehungsnetzwerke verschoben [6, S. 90–105, 7, S. 11, 15, 32, 8, S. 87–96]. So sind die breite Kritik an der Stellungnahme der Bundesregierung [9] von BAGSO [10], DigiBÄM [11] und der Stiftung Digitale Chancen [12] und die darin enthaltenen Forderungen an die Bundesregierung, Rahmenbedingungen zum Ausbau digitaler Unterstützungsstrukturen für ältere Menschen auf Bundesebene sowie konkrete Maßnahmen zu schaffen, nach wie vor aktuell. Hier lässt sich eine Parallele zwischen der Perspektive auf Pflegebedarf und Digitalisierung aufzeigen. Die oberflächliche Fokussierung auf eine kompetenzorientierte Perspektive auf älterer Menschen bei gleichzeitiger Postulierung, dass ältere Menschen nur ausreichend motiviert werden müssten, individualisiert nicht nur die Verantwortung für die Digitalisierung des Einzelnen, sondern verhindert auch Erkenntnisse darüber, welche Gruppen älterer Menschen besondere Hürden zu überwinden haben und deswegen besonderer Unterstützung bedürfen [12, S. 10].

Trotz weiterer struktureller Veränderungen, die durch das „Digitale-Versorgung-Gesetz“ [13] und das „Digitale-Versorgung-und-Pflege-Modernisierungs-Gesetz“ [14], die zunehmend alltagsweltliche digitale Kompetenzen auf individueller Ebene erfordern, herbeigeführt werden sollen, erwähnt das SGB V [15] in § 20k zwar die Notwendigkeit, die digitalen Übergänge aller Gruppen zu gewährleisten, schafft dafür aber keine konkreten Rahmenbedingungen. Diese bleiben auch im SGB XI unerwähnt, wo die Digitalisierung des Pflegesystems u. a. in § 8, § 40a, § 148 geregelt ist [5]. Der Subtext zur zunehmenden Digitalisierung zwischen Staat und Bürger fokussiert auf die Vorteile für ältere Menschen, durch die Digitalisierung lange ein selbstbestimmtes und unabhängiges Leben in der eigenen Wohnung führen zu können. Die Alltagsrealität und die digitalen Kompetenzen der zunehmend heterogenen Gruppe älterer Menschen bleiben dabei unberücksichtigt. Darüber hinaus wird die Eigenverantwortung, ein unabhängige*r Bürger*in zu bleiben und nicht in die Verantwortung des Staates zu geraten, betont. Die Heterogenität der älteren Bevölkerung und Ungleichheiten innerhalb der Gruppe Älterer werden in den analysierten Rechtsdokumenten nicht berücksichtigt.

### Alter und Digitalisierung als Leerstelle in politisch bindenden Dokumenten

Die Dokumente von zivilgesellschaftlichen Organisationen, die sich dezidiert mit Alter auseinandersetzen, kritisieren eine digitale Kluft zwischen den Generationen und benennen Gründe, warum ein Teil der älteren Menschen keine digitalen Geräte verwendet. Darüber hinaus werden bestehende Unterstützungsbedarfe benannt sowie Lösungsansätze und Handlungsempfehlungen formuliert [16]. Demgegenüber wird in nichtaltersspezifischen Politikdokumenten, wie dem Koalitionsvertrag, keine digitale Spaltung zwischen älteren und jüngeren Generationen thematisiert und stattdessen eine digitale Zukunftsstrategie mit dem Fokus auf Kinder, Schule, Bildung und Ausbildung skizziert, in der seniorenfreundliche Ansätze im anvisierten digitalen Raum erwähnt werden [18, S. 102]. Um die digitale (Alters‑)Kluft zu verringern, werden in der Folge nur wenige Programme angeboten und weniger Ressourcen investiert (im Vergleich zu den Investitionen im schulischen Bildungsbereich). Diese fehlende Strategie zur Sicherstellung der Integration älterer Menschen in die Digitalisierung wird in Dokumenten von zivilgesellschaftlichen Akteuren auf Bundesebene kritisiert [16, S. 1–5, 17, S. 4–14]. Die bisherigen und aktuellen politischen Maßnahmen zur Digitalisierung stellen ihrer Ansicht nach wirtschaftliche Faktoren und nicht den Abbau von Ungleichheiten in den Vordergrund. Die Stiftung Digitale Chancen sowie DigiBÄM kritisieren, dass Bevölkerungsgruppen, die keinen einfachen Zugang zur Digitalisierung haben, ignoriert werden, und dass Empfehlungen und Forderungen an die Bundesregierung, dies zu ändern, seit mehr als 20 Jahren ungehört geblieben sind [12, S. 4 f.]. Die Analyse zeigt jedoch, dass Ungleichheiten innerhalb der Gruppe älterer Menschen beispielsweise aufgrund von Geschlecht, Alter, Bildung, sozioökonomischem Hintergrund, ländlichem oder städtischem Lebensstil und Ethnie auch in den Dokumenten der zivilgesellschaftlichen Organisationen auf Bundesebene eine untergeordnete Rolle spielen. Die Kluft zwischen den Forderungen der Expert*innengruppen mit den Schwerpunkten Altern, ältere Menschen und (digitale) Technologien [10, 11, 16, 17] und den vom Bund und Ländern finanzierten Maßnahmen und Programmen lässt sich deutlich anhand des Digitalpakts Alter zeigen. Während die Expert*innengruppen flächendeckende, standardisierte und niedrigschwellige Anlaufstellen und Unterstützungsangebote fordern, die sich speziell an ältere Menschen richten, ist der Digitalpakt Alter bisher die einzige konkrete Umsetzung dieser Forderungen. Der Digitalpakt Alter [19] wurde als Reaktion auf den Achten Altersbericht der Bundesregierung durch die BAGSO ins Leben gerufen und vom Bundesministerium für Familie, Senioren, Frauen und Jugend im Jahr 2021 als zeitlich und finanziell stark begrenztes Modellprojekt finanziert, das 2023 um 2 Jahre verlängert wurde. Während die zivilgesellschaftlichen Akteure betonen, dass neben ehrenamtlichen Strukturen, die auf Peer-Learning basieren, bezahltes Personal zum Aufbau nachhaltiger Strukturen unerlässlich sei, verlässt sich eine solche begrenzte Finanzierung mit kurzen Programmlaufzeiten weiterhin auf ehrenamtliches Engagement. Damit ist die Nachhaltigkeit des Digitalpakts Alter infrage gestellt.

Die politische Strategie, wenig finanzielle Ressourcen in die Digitalisierung älterer Menschen zu investieren, läuft einer Entwicklung zu einer zunehmend digitalisierten Gesellschaft entgegen. So finden wichtige Themen wie Datenschutz und ethische Fragen wenig Aufmerksamkeit. Dies kann zur Folge haben, dass im Politischen und auch im Privaten zunehmend über die Köpfe der älteren Nutzer*innen hinweg entschieden wird [17, S. 13].

## Diskussion

Die kritische Analyse fördert zwei zentrale Erkenntnisse zutage: Zum einen ist dies die Individualisierung gesellschaftlicher Probleme im Bereich Sorgebeziehungen. Altern wird primär im Kontext des demografischen Wandels und damit als eine Herausforderung für die Gesellschaft dargestellt. Diese Problematisierung führt aber nicht dazu, Digitalisierung und Altern zum investitionswürdigen Ziel von Sozialpolitik zu machen. Vielmehr werden Lösungsansätze, die auf privaten Beziehungs- und Sorgenetzwerken sowie ehrenamtlichem Engagement basieren, entwickelt. Die staatliche Unterstützung folgt dem Subsidiaritätsprinzip und dem Prinzip der Selbstsorge. Dieser individualisierende Lösungsansatz stößt durch sich verändernde Familienstrukturen, Geschlechter- und Arbeitsverhältnisse und die demografische Veränderung der Bevölkerungszusammensetzung bereits an seine Grenzen und wird sich in Zukunft weiter zuspitzen. Für diese Problematik wird die Digitalisierung als adäquate Lösungsoption gehandelt. Sie soll sowohl die Selbstständigkeit von älteren Personen fördern und ausdehnen als auch private Sorgebeziehungen über räumliche Distanzen hinweg ermöglichen. Dabei wird der zunächst entstehende erhöhte Sorgebedarf durch die notwendige Unterstützung in der Handhabung digitaler Geräte ebenfalls an die persönlichen Sorgenetzwerke delegiert. Von älteren Personen wird ein aktives Bemühen um die eigene Digitalisierung erwartet, ohne sie aktiv in die digitalen Entwicklungen miteinzubeziehen, ihnen Entscheidungskompetenzen einzuräumen oder das Erlernen langfristig finanziell sicherzustellen. Sie werden zudem als freiwillig Arbeitende zur Peer-Unterstützung anderer Älterer im Bereich Digitalisierung angerufen.

Zum anderen zeigt die Analyse die Diskrepanz zwischen den beiden Ebenen der analysierten Dokumente auf, die zum einen konkrete rechtliche Handlungsrahmen schaffen, wie Sozialgesetze [5, 13–15] oder der Koalitionsvertrag [18], und denen, die aus Praxiserfahrung entstehen, wie Positionspapiere und Politikberichte zivilgesellschaftlicher Organisationen [3, 4, 9, 11, 16, 17, 20]. Die Sozialgesetzgebung versteht Digitalisierung in den Bereichen Gesundheit, Pflege und Versorgung als Chance und treibt sie voran. Der Schnittmenge von Digitalisierung und Altern wird jedoch eine geringe Priorität eingeräumt, was sich in oberflächlicher Darstellung und dem fehlenden rechtlichen Handlungsrahmen widerspiegelt. Auf der Ebene der zivilgesellschaftlichen Dokumente wird das Zusammenspiel in verschiedene Richtungen diskutiert und mehr Engagement des Staates gefordert, was nur bedingt auf Widerhall stößt, wie beispielsweise der Digitalpakt Alter zeigt. Eben diesen bräuchte es jedoch, damit die Digitalisierung ihre positiven Effekte gerade für die heterogene Gruppe der älteren Menschen stärker entfalten könnte.

## Fazit für die Praxis

- Die strukturellen Veränderungen in Form von Gesetzen zum Ausbau von Digitalisierung machen die Notwendigkeit, alltagsweltliche digitale Kompetenzen alter Menschen zu fördern und Anlaufstellen bei Fragen zu schaffen, unabdingbar.
- Die stetig wachsende Bevölkerung der alleinlebenden Menschen im Alter und v. a, derer ohne Sorgenetzwerke sollte stärker in den Fokus der Sozialpolitik gerückt werden.
- Eine Bereitstellung stabiler und niedrigschwelliger Unterstützungssysteme für Menschen, die nur geringfügig online oder offline sind (und es bleiben wollen), gewährleistet die Erfüllung von Bürgerrechten und -pflichten.
- Die kritische Dokumentenanalyse eignet sich als Methode, implizite Perspektiven auf Altern und Digitalisierung im Bereich von Sozialpolitik sichtbar zu machen und Handlungsbedarfe aufzuzeigen.
- Die Heterogenität der älteren Bevölkerung und Ungleichheiten innerhalb der Gruppe Älterer müssen auf rechtlicher Ebene stärker berücksichtigt werden.

## Footnotes

1 Sorge wird hier in Anlehnung an den im englischsprachigen Diskurs geläufigen Begriff „Care“, der eine Bandbreite von Tätigkeiten wie betreuen, pflegen, versorgen, unterstützen, sich kümmern und zuwenden umfasst, genutzt. Damit greifen wir Sorgebeziehungen ebenso weit und beschränken diese nicht auf private und professionelle Pflegearrangements.

2 Die Berichte auf Landesebene beziehen sich auf Hessen.

## References

[1] Bacchi CL (2009) Analysing policy: what’s the problem represented to be? Pearson

[2] Schneider A, Ingram H (1993) Social construction of target populations: implications for politics and policy. Am Polit Sci Rev 87(2):334–347. 10.2307/2939044

[3] Bundesministerium für Familie, Senioren, Frauen und Jugend (2016) Siebter Altenbericht – Sorge und Mitverantwortung in der Kommune – Aufbau und Sicherung zukunftsfähiger Gemeinschaften. Berlin. https://www.bmfsfj.de/resource/blob/120144/2a5de459ec4984cb2f83739785c908d6/7-altenbericht-bundestagsdrucksache-data.pdf

[4] Bundesministerium für Familie, Senioren, Frauen und Jugend (2020) Achter Altersbericht: Ältere Menschen und Digitalisierung. Berlin. https://www.bmfsfj.de/resource/blob/159916/9f488c2a406ccc42cb1a694944230c96/achter-altersbericht-bundestagsdrucksache-data.pdf

[5] Sozialgesetzbuch (SGB) XI. Pflegeversicherung. Berlin. Deutscher Bundestag.

[6] Hessische Ministerin für Digitale Strategie und Entwicklung (2021) Digitales Hessen: Wo Zukunft zuhause ist. Wiesbaden. https://digitales.hessen.de/sites/digitales.hessen.de/files/2023-02/01_pdf_digitalstrategie_gesamt_barrierefrei.pdf

[7] Deutsche Bundesregierung (2021) Digitalisierung gestalten. Umsetzungsstrategie der Bundesregierung. Berlin. https://www.bundesregierung.de/resource/blob/992814/1605036/ad8d8a0079e287f694f04cbccd93f591/digitalisierung-gestalten-download-bpa-data.pdf

[8] Hessische Landesregierung (2016) Strategie Digitales Hessen. Intelligent. Vernetzt. Für Alle. Wiesbaden

[9] Bundesministerium für Familie, Senioren, Frauen und Jugend (2020) Achter Altersbericht: Stellungnahme der Bundesregierung. Berlin. https://www.bmfsfj.de/resource/blob/159916/9f488c2a406ccc42cb1a694944230c96/achter-altersbericht-bundestagsdrucksache-data.pdf

[10] Bundesarbeitsgemeinschaft der Seniorenorganisationen (2020) Achter Altersbericht Stellungnahme BAGSO: Ältere Menschen und Digitalisierung. Bonn. https://www.bagso.de/fileadmin/user_upload/bagso/06_Veroeffentlichungen/2020/BAGSO-Stellungnahme_Achter_Altersbericht_Digitalisierung.pdf

[11] Fachbeirat Digitalisierung und Bildung für ältere Menschen (DigiBÄM) (2020) Bildung als Wegweiser in eine humane digitale Gesellschaft! Stellungnahme des Fachbeirats „Digitalisierung und Bildung für ältere Menschen“ zum Achten Altersbericht der Bundesregierung. https://www.achter-altersbericht.de/fileadmin/user_upload/Stellungnahme_FB_Digi_und_Bildung_fuer_aeltere_Menschen.pdf

[12] Kubicek H (2020) Positionspapier: Soziale Ungleichheit durch Zugang für alle reduzieren – Chance vertan. Kritik am Achten Altersbericht zu den Ausführungen zum Thema Ältere Menschen und Internet. Stiftung Digitale Chancen, Berlin. https://www.achter-altersbericht.de/fileadmin/altersbericht/pdf/Stellungnahmen/Stellungnahme_Prof_Kubicek_Stiftung_Digitale_Chancen.pdf

[13] Digitale-Versorgung-Gesetz (DVG). Deutscher Bundestag, Berlin

[14] Digitale-Versorgung-und-Pflege-Modernisierungs-Gesetz (DVPMG). Deutscher Bundestag, Berlin

[15] Sozialgesetzbuch (SGB) V. Gesetzliche Krankenversicherung. Deutscher Bundestag, Berlin

[16] Bundesarbeitsgemeinschaft der Seniorenorganisationen (2017) Positionspapier: Ältere Menschen in der digitalen Welt. Bonn. https://www.bagso.de/fileadmin/user_upload/bagso/06_Veroeffentlichungen/2020/BAGSO_Positionspapier_Aeltere_Menschen_Digitale_Welt.pdf

[17] Fachausschuss „Alter und Technik“ (2022) Positionspapier: Den digitalen Wandel im höheren Lebensalter in Deutschland gestalten – Jetzt oder nie: ein Positionspapier des FA Alte und Technik der DGGG. https://fa-alter-technik.de/wp-content/uploads/2022/06/2022-06-21_Finalversion_Langtext.pdf

[18] Sozialdemokratischen Partei Deutschlands (SPD), BÜNDNIS 90/DIE GRÜNEN, Freie Demokraten (FDP) (2021) Koalitionsvertrag 2021–2025: Mehr Fortschritt wagen – Bündnis für Freiheit, Gerechtigkeit und Nachhaltigkeit. Berlin. https://www.spd.de/fileadmin/Dokumente/Koalitionsvertrag/Koalitionsvertrag_2021-2025.pdf

[19] Digitalpakt Alter (2021) Digitale Teilhabe ist gesellschaftliche Teilhabe. Hannover/Bonn. https://www.digitalpakt-alter.de/gemeinsame-erklaerung/

[20] Bundesministerium für Familie, Senioren, Frauen und Jugend (2016) Siebter Altenbericht: Stellungnahme der Bundesregierung. Berlin. https://www.bmfsfj.de/resource/blob/120144/2a5de459ec4984cb2f83739785c908d6/7-altenbericht-bundestagsdrucksache-data.pdf

